# Entrepreneurial efforts and opportunity costs: evidence from twitch streamers

**DOI:** 10.1007/s11365-023-00849-2

**Published:** 2023-04-15

**Authors:** Philip Wollborn, David Dornekott, Ulrike Holder

**Affiliations:** 1 Institute of Strategic Management, University of Muenster, Leonardo-Campus 18, Muenster, 48149 Germany; 2 Department of Marketing, University of Osnabrueck, Osnabrueck, Germany

**Keywords:** Digital entrepreneurship, Content creation, Self-employment, Opportunity costs, Platform work, Live streaming, COVID-19, Difference-in-differences

## Abstract

With the rise of digital platforms, individuals’ possibilities to generate income have increased drastically. In this context, we present digital content creation as a form of (digital) entrepreneurship that is characterized by potentially high but also uncertain revenues. As the cost structure of content creation mostly depends on opportunity costs, it stands in contrast to other popular platform-work options. We demonstrate how a stark and unexpected reduction in opportunity costs affects the actual decision to produce digital content. Exploiting the first wave of the COVID-19 pandemic, we measure how individuals (streamers) who operate on a live streaming platform, respond to a sudden change in external factors while accounting for individual differences in initial conditions. We observe intensified efforts across the spectrum of streamers and find particularly strong reactions from newcomer streamers. We further show that only the most successful newcomers sustain their increased efforts even when opportunity costs start to rise again. Our results are consistent with the initial assumption that an individual’s decision on taking up or intensifying entrepreneurial efforts on digital platforms is strongly affected by their opportunity costs. The results further imply that there is a large potential in individuals who might be willing to become entrepreneurs but are restricted by external conditions. As platform-based digital entrepreneurship offers high flexibility and very low entry barriers, measures for lowering opportunity costs could therefore help to unleash this potential. To maintain a steady influx of new talents, content platforms should increase their support for smaller creators and policymakers should provide easily accessible platforms to ease the way into entrepreneurship for these individuals.

## Introduction

In the last decade, the number of digital platforms, as well as individuals deriving income through them, has grown substantially. As these platforms provide individuals with highly flexible and independent work opportunities (Hall & Krueger, 2018), extant research on platform work has focused on its potential to generate income in reaction to fluctuations in employment (Fos et al., 2021; Jackson, 2020; Koustas, 2018), as well as its ability to enable entrepreneurial activity outside of the platform work itself (Barrios et al., 2020; Burtch et al., 2018). A type of platform work that so far has been overlooked in the entrepreneurship literature, however, is digital content creation, which is the act of creating and publishing digital media, such as videos, images, audio files, or text documents. Compared to the activities that are typically studied in the literature (e.g. e-commerce, gig work, room sharing), content creation differs strongly from these activities in multiple characteristics, making it especially interesting for entrepreneurship research. First, in content creation, individuals have large a-priori uncertainty around the possession of assets necessary to generate income from it. Without actually trying it, content creators do not know if their content will generate sufficient demand, whereas e.g. supplies for ridesharing, food delivery, craftsmenship, or room sharing services can anticipate whether they operate in an area of sufficiently high demand fairly well. Second, the aforementioned activities only have limited growth potential, which is why often they are seen more as a flexible full-time-job equivalent enabling other entrepreneurial activities (Barrios et al., 2020; Burtch et al., 2018). Content creation, on the other hand, offers very large (albeit highly uncertain) potential payoff (D’Anastasio, 2021; Needleman, 2021; Twitch.tv, 2021). Therefore, it stands in stark contrast to the given examples of gig work, which instead, provide immediate monetary return proportional to time and effort invested. Third, in the digital content creation industry, production costs mostly only vary with the content creator’s own efforts but are constant in regard to the demand side as platforms bear most of the variable costs (e.g. server costs), whereas income can grow disproportionately with audience size. Thus, marginal costs are mostly only dependent on time spent creating content while other platform work (e.g. room sharing, e-commerce) face much higher marginal costs when existing capacities are maxed out. However, digital content creation often requires more up-front investments in terms of opportunity costs (as we will explain in more detail later on) than other platform work until individuals can actually generate income.

In our view, content creation therefore resembles an interesting area to study from an entrepreneurial and quantitative perspective, especially as existing literature to the entrepreneurial side of the topic chose mostly qualitative approaches (Ashman et al., 2018; Johnson & Woodcock, 2019a, b; Mardon et al., 2018; Törhönen et al., 2020, 2021). In this context, we study how individuals’ efforts to create content (controlling for differences in initial conditions) change in response to a stark and unexpected change in opportunity costs.

According to the utility maximization model for career choice by Douglas and Shepherd (2000), individuals choose to either be employed or engage in entrepreneurial activities, depending on a set of factors like attitudes towards work effort or risk taking. Now suppose an external shock leads to a sudden and unforeseen change of these conditions, resulting in a higher tolerance of work effort or a lower risk associated with the option to become an entrepreneur. Some individuals that ex ante chose to be employed should, all other things being equal, thus receive a higher utility through pursuing entrepreneurial activities after the shock. Furthermore, when said conditions return to pre-shock levels, new entrepreneurs might choose to (not) return to being employed if the expected utility turns out to be lower (higher) than initially thought. In terms of the model by Douglas and Shepherd (2000), such a result can be explained by a reduction in uncertainty. Within this framework, a content creator considering professionalization will base their decision to increase efforts both on positive signals of potential future *benefit* (i.e. in the form of increased audience), as well as the *costs* associated with such efforts. As digital content creation’s capital requirements^1^ are typically negligible for residents of industrialized nations, the main cost associated with increased activities are of opportunity, mainly in the form of time that could have been invested towards other ends, such as employment work, leisure or educational attainment. As previously argued in the literature, lower opportunity costs are more likely to encourage individuals to engage in entrepreneurial activities (Agrawal et al., 2018; Amit et al., 1995; Berkhout et al., 2011) and we would thus expect the same to be true for content creators. However, most studies typically use variables of nominal nature or that have high preconditions (employment status, firm founding, patenting) to observe entrepreneurial activity. Similarly to Burtch et al. (2018), who use volume and rate of crowdfunding campaigns, we leverage different metrics of time devoted to content creation as a more fluid way to measure entrepreneurial activity. This has the advantage that we are able to observe increases in activity even if they are scaled back before e.g. a firm would have been founded. Thus, we also measure attempts to increase activity that would have been overlooked otherwise.

To analyze such behavioral changes, we exploit the sudden changes and restrictions brought about by the first wave of the COVID-19 pandemic using a difference-in-differences (DiD) design. Worldwide, the pandemic was first met with a reduction in mobility by staying home and changes in consumption behavior, especially regarding leisure activities (van Leeuwen et al., 2020). Concurrently, shutdowns in certain business areas and supply-chain disruptions distressed the economy, resulting in dramatic short-term effects on employment, while schools and universities temporarily closed. As such, we posit that the COVID-19 pandemic and related containment measures were an unexpected, positive shock to individuals’ available time, while simultaneously decreasing other opportunities to derive income, including gig work (Ivaldi & Palikot, 2020), resulting in overall lowered opportunity costs for entrepreneurial activity on purely digital content platforms. That in turn, should enable an overall increase of entrepreneurial efforts on such platforms according to the utility maximization model for career choice by Douglas and Shepherd (2000) and the *External Enablers* framework by Davidsson et al. (2020).

This study provides empirical evidence for Douglas and Shepherd’s (2000) career choice model and *External Enablers* framework (Davidsson et al., 2020) based on observed behavioral changes in efforts while controlling for differences in individuals’ initial conditions. Furthermore, we apply a new way to measure entrepreneurial activity in a setting where the decision to (increasingly) engage in entrepreneurial activity is less a question of yes or no, but instead allows for more fluid and less predefined ways into entrepreneurship than “traditional entrepreneurship” does (Nambisan, 2017). Furthermore, the study addresses content creation as a form of (digital) entrepreneurship in contrast to other platform work, which typically is much more capacity-constrained (e.g. ride sharing, craftsmen services, freelance work) and therefore offers relatively plane growth paths making such activities more comparable to employment-like conditions instead (Kraus et al., 2018; Kuhn & Maleki, 2017; Sussan & Acs, 2017).

The next section provides a theoretical background for our considerations and assumptions. "Methodology" section then provides more details on the live streaming platform Twitch and streamers’ range of action and monetization options. In "Data and descriptive statistics" section, we describe our data and empirical strategy, the results of which we present in "Empirical analysis" section. We conclude with a discussion and final remarks in "Discussion" and "Concluding remarks" section.

## Theoretical background – opportunity costs and the pandemic as external enablers for entrepreneurial efforts

The question why some individuals choose to be entrepreneurs while others choose to be employed has been present in the academic literature since around the 1990s (Baumol, 1990; Campbell, 1992; Douglas & Shepherd, 2000; Evans & Leighton, 1990). While utility maximization has been presented as an explanation early on (Baumol, 1990), the question what exactly affects individuals’ utility quickly followed and is still studied to this day. Personal attitudes such as risk aversion (Campbell, 1992; Douglas & Shepherd, 2000) and external characteristics like working conditions (Douglas & Shepherd, 2000; Eisenhauer, 1995) or (expected) income from employment vs. self-employment work (Berkhout et al., 2011; Douglas & Shepherd, 2000; Millán et al., 2012; Svaleryd, 2015) quickly followed to explain individuals’ utility functions. Furthermore, there is a deep research stream regarding entrepreneurial intent (see Schlaegel & Koenig, 2014 for a meta-analysis). However, while entrepreneurial intent varies strongly between individuals, this alone does not explain why e.g. some individuals choose stay employed even when entrepreneurial intent is high (Douglas & Shepherd, 2000). The Douglas and Shepherd (2000) model for career choice presents an individual’s choice between employment and self-employment^2^ for a given period as a function of income, work effort, risk, independence, and other working conditions in that period. The Douglas and Shepherd (2000) further state, that the employment decision can vary between periods (e.g. an individual leaning towards entrepreneurship might lack opportunity to become an entrepreneur at one moment but chooses differently at a later point in time). Thus, when certain conditions change – be it suddenly or gradually – one would expect some individuals to change their behavior as reaction. Taking up on this idea, the external enablers framework by Davidsson (2015) and Davidsson et al., (2020, 2021) provides a very fitting narrative on how a change in external circumstances might spark new venture creation processes as they provide new or enhanced opportunities for certain business models. The authors describe their framework as providing.

“Structure and terminology for analyzing the enabling effects of different types of external change for entrepreneurial initiatives, such as technological breakthroughs, regulatory reforms, macroeconomic shifts, demographic sociocultural trends, and changes to the natural environment” (Davidsson et al., 2021, p. 2).

**Fig. 1:**
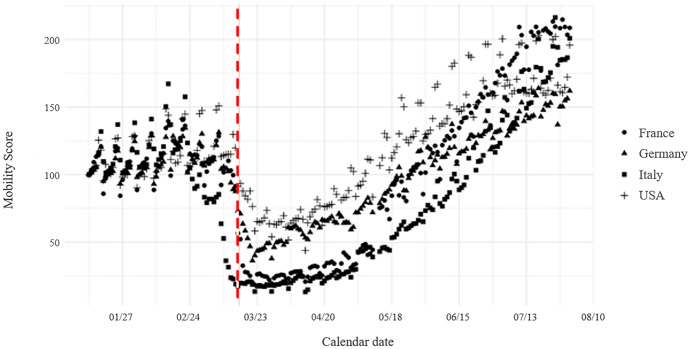
Mobility Score (January – August 2020). Note: To quantify changes in mobility over the observed time frame, we retrieved mobility data provided by Apple and Google via the *covdata* package for R (Healy, 2020)

When the COVID-19 pandemic became omnipresent in March 2020, individuals and politics reacted by strongly decreasing individual mobility as can be seen in Fig. 1. In terms of the *External Enabler* framework, voluntary changes in everyday behavior (decreased demand for physical social interactions and increased demand for home-based digital activities) constitute *sociocultural trends*, while political measures such as curfews or complete shutdowns of certain businesses (bars and restaurants, live entertainment, commercial sports activities) are *regulatory reforms*.

In this context, the beginning of the COVID-19 pandemic therefore constitutes an External Enabler (Davidsson et al., 2021) that affected individuals’ utility functions (Douglas & Shepherd, 2000) by lowering the risk involved and the perceived disutility of work effort and other working conditions in self-employment as well as increasing the expected income from it. In our view, these changes effectively lowered individuals’ opportunity costs of increasing entrepreneurial activity, especially if such efforts can be conducted from home, as is the case in digital content creation.

### Digital content creation as entrepreneurship

Another question that needs to be discussed is, whether digital content creation qualifies as entrepreneurial activity. Typically, entrepreneurship has been described as taking on the risk of “producing a product that would sell for more than the cost of production.” (Douglas & Shepherd, 2000, p. 232) or as responding to opportunities enabled through technological advancements with new services or products (Baumol, 1990; Douglas & Shepherd, 2000; Holmes & Schmitz, 1990). Professional content creators operate exactly like that: be it bloggers, YouTubers, social media influencers, or – as in in this study – live streamers. Such content creators need to take on the risk of producing their content before knowing whether there is demand for it. More precisely, becoming a successful content creator requires an unknown investment of time and resources, as well as an initial endowment with abstract characteristics related to ability and personality traits and potential content creators only have a limited ability to ascertain their success potential without first attempting their luck. Furthermore, demand alone often does not suffice, as the produced content does not only need to be enjoyable but also monetizable. However, the typical ecosystem provided by content platforms is that the content needs to be free of charge for consumers and can only be monetized subsequently either directly through consumer payments or through third party payments such as sponsors.^3^ In our study, we focus on live video streaming (henceforth live streaming), which is the practice of broadcasting digital video content produced in real-time over the internet, on the platform Twitch^4^ as a prime example for the professional content creation industry.

The live streaming industry is one of the largest content industries and is mostly devoted to entertainment, informational, and educational content. In 2014, market leader Twitch was bought by Amazon for 970 million U.S. dollars and overall market size in terms of revenue was estimated to 9.3 billion U.S. dollars in 2020 (SuperData, 2021). Furthermore, successful live streamers are able to generate sizeable incomes: A 2021 leak of payouts by Twitch (Needleman, 2021; Twitch.tv, 2021) to the top ten thousand streamers by revenue revealed median monthly payouts of 1,665$, rising to 49,821$ for the 100 largest streamers and 155,323$ for the top ten and these direct payments do not include any external revenue sources (such as payments from promotional activities) that make up a far greater share of a streamer’s total income (D’Anastasio, 2021).

In our view, the aforementioned characteristic of digital content creation qualify the activity as a form of entrepreneurship (Ashman et al., 2018; Johnson & Woodcock, 2019a, b; Mardon et al., 2018; Törhönen et al., 2021), wherein initially high outcome uncertainty is gradually reduced through market feedback (in this case resonance with viewers) and attempts at achieving product-market-fit (i.e. through adapting content to accommodate the demands of certain types of viewers).^5^ Furthermore, content creation also fulfills the criteria of the more recent term “digital entrepreneurship”, commonly described as the act of revenue generation from digital goods (Guthrie, 2014) or any kind of venture-related activities that requires the use of digital technologies (Sussan & Acs, 2017). In this context, we want to stress the distinction of content creation to other practices of online-related work such as operating as driver on ridesharing or food-delivery services. Such activities might also qualify as digital entrepreneurship according to the provided definitions, but actually lack the potential to become more than an income opportunity equivalent to a full-time equivalent income. Though such gig work still is self-employed work, it has often been viewed not as entrepreneurship, but as a form of income insurance (Garin et al., 2020; Koustas, 2018) that complements actual entrepreneurial activities (Barrios et al., 2020; Burtch et al., 2018).

We argue that the increase in individuals’ available time and decrease in employment opportunities can be viewed as a decrease to opportunity costs for increased entrepreneurial effort. This should especially be true for entrepreneurial activities that can be conducted entirely from home such as live streaming. In the literature, opportunity costs have often been described purely from the perspective of foregone (future) earnings from paid employment (Amit et al., 1995; Berkhout et al., 2011; Burtch et al., 2018; Cassar, 2006; Nikolaev et al., 2018). In these studies, reduced opportunity costs were found to have a positive impact on entrepreneurial activities. Additionally, under- and unemployment were also shown to be positively correlated with entrepreneurship options (Block & Koellinger, 2009; Burtch et al., 2018; Storey, 1991) or self-employment (Fossen, 2020; Thurik et al., 2008). Being another critical and constrained resource, available time has recently also been studied from the perspective of opportunity costs and was found to positively affect entrepreneurial activity (Agrawal et al., 2018; Burtch et al., 2018). These results also fall in line with the implications of the model for career choice by Douglas and Shepherd (2000), in which an increase in available time and a reduced set of other job opportunities eases the disutility that results from increased work effort and lowers the perceived risk when choosing entrepreneurship instead of employment work. Furthermore, Törhönen et al. (2020) showed that time spent streaming is significantly correlated with the impression it being helpful to generate income and improve the career development of individuals, underlining the potential of this variable as measurement for entrepreneurial efforts.

**Fig. 2:**
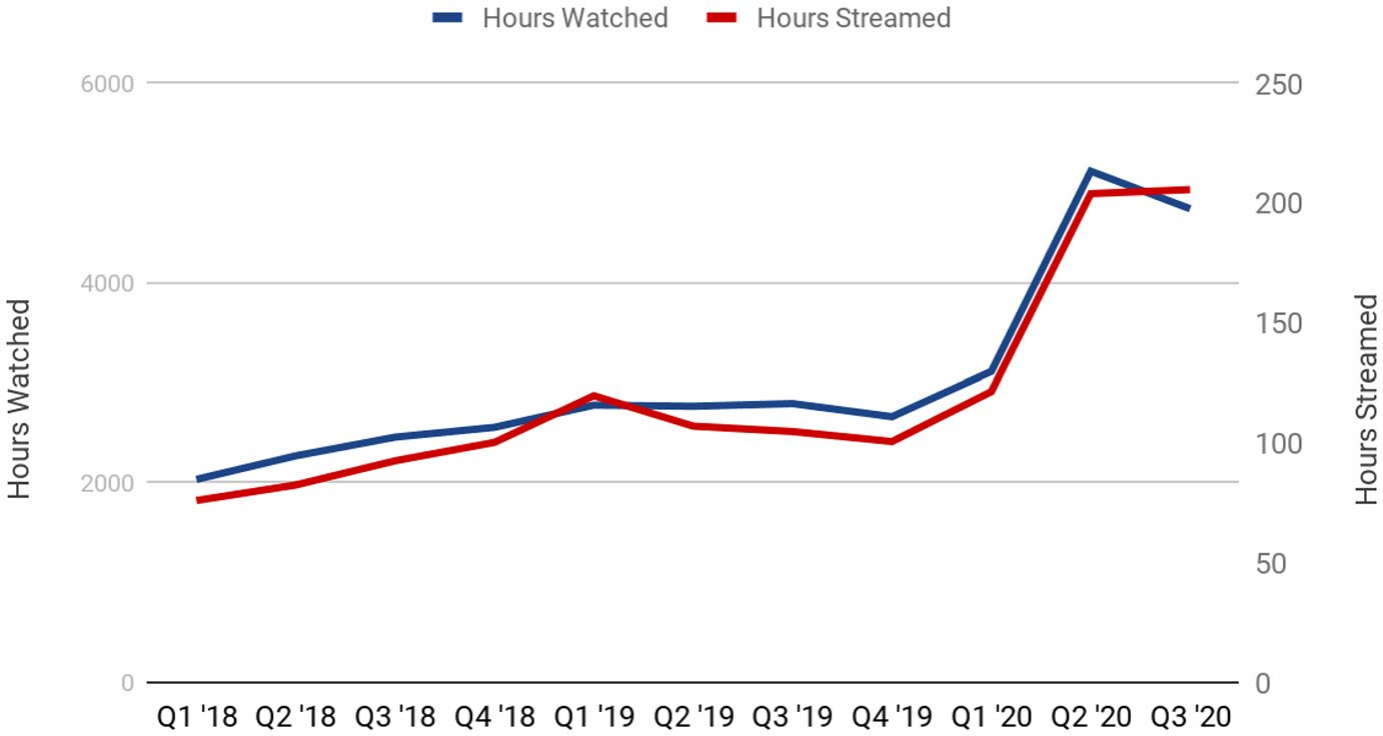
Twitch User Metrics (2018 – 2020). Note: Data derived from Streamlabs (2020a, c)

Figure 1 demonstrates that individual mobility heavily decreased in the beginning of the COVID-19 pandemic, which lead to people staying at home and experiencing fewer in-person social interactions. In addition to the aforementioned arguments for opportunity costs, Törhönen et al. (2020) find that when live streaming is seen as beneficial for social interactions, this was found to increase the intention to create video content as well as the actual time invested per week. Beyond that, with the start of the pandemic, there also was a strong and sudden increase in demand (see Fig. 2), which, at least in parts, should be connected with the social elements of live streaming. Given the possibilities for interactions during streams (e.g. via chat) and the feeling of co-presence the medium can provide (Diwanji et al., 2020), the strong increase in viewership can likely be attributed to a need for social interactivity. Therefore, the increase in demand presented an opportunity for both incumbent streamers to attract new viewers, as well as for new streamers to enter the market with a higher potential for success than before. Furthermore, times of economic hardship can have an increasing effect on entrepreneurial activity (Amorós et al., 2019; Huang et al., 2020) and in this regard, the pandemic had a drastic impact on the global economy with entire industries being shut down (e.g. hospitality, tourism) and a strong increase in unemployment rates (Adams-Prassl et al., 2020; Alstadsæter et al., 2020; Bauer & Weber, 2020; Bick & Blandin, 2020; Cho & Winters, 2020; Davidsson et al., 2021; Juranek et al., 2020). Thus, we posit that the pandemic and the related containment measures were an unexpected, exogenous shock to individuals’ amount of idle time spent confined to their home as well as their available income that lowered individuals’ opportunity costs to increase entrepreneurial activity in the form of digital content creation.

## Methodology

There are several studies that have explored the motivations of streamers and viewers (e.g., Gros et al., 2017; Johnson & Woodcock, 2019a, b; Sjöblom & Hamari, 2017; Törhönen et al., 2020; Wulf et al., 2020) as well as how popular streamers cultivate their fanbases (e.g., Gandolfi, 2016; Sjöblom et al., 2019). In general, streamers are highly heterogeneous in the intensity of their activity and aspirations for economic success, ranging from pure hobbyists with no expectations of monetary reward to fully professionalized individuals deriving the entirety of their income from streaming. To provide a better understanding for live streaming as entrepreneurship, our estimation strategy, and how we distinguish streamers with entrepreneurial ambition from pure hobbyists, we want to present the platform Twitch and the income generation process on the platform a bit more deeply.

As laid out above, Twitch currently dominates the market for live streaming. From February to March 2020, Twitch recorded an increase of total hours watched by almost 23% and an enormous increase of the broadcasting user base, by about 64% (Streamlabs, 2020a). Figure 2 shows the overall development of streaming hours watched and hours streamed on Twitch from 2018 until 2020. Poignantly, viewing behavior on Twitch during that period seems to be inversely correlated with the mobility data as provided in Fig. 1. With several countries going into lockdown in March 2020, hours watched on Twitch increased, reaching their peak in April, after which they stabilized at a slightly lower level in May and June, when mobility again increased in most countries. These stabilized viewer levels were still roughly 50% higher than at the beginning of 2020, suggesting that Twitch has benefited from the COVID-19 pandemic in a sustainable way.

The growth in live streaming during a global pandemic was likely abetted by its unique mixture of features. In addition to the purely consumptive act of watching live-streamed content, live streaming’s interactive features add social elements. Live streaming embodies a socially enjoyable experience and an easily accessible space to interact with a community (Diwanji et al., 2020; Törhönen et al., 2020; Wulf et al., 2020), which might be more highly sought after in times of social distancing.

Turning to the supply side of streaming, a comparatively small fraction of streamers relies on a range of different monetization options to generate income on the platform. In Table 1, we present the most common revenue sources for live streamers operating on the platform Twitch. Most of these revenue sources however, can also be found on other major content platforms.^6^ On Twitch, the most prominent revenue sources are monthly subscriptions, “donations”^7^ and advertisement. In addition, and with rising popularity of individual streamers, other options such as sponsored content and merchandising can provide further sizeable external revenue sources.

**Table 1:** Revenue Sources for Streamers

	Internal	External
One-time payments	virtual currency gifted subscriptions (donations) referral links merchandise	money transfers coupon codes referral links merchandise
Recurring payments	subscriptions	digital patronage
Sponsoring	paid content advertisement revenue share	paid content promotional activities event/appearance salaries

Source: Own research based on observations on Twitch.tv

In 2019, the internal options stood open to up 657.504 so-called “affiliate” streamers as well as 29.707 “partners”. The opportunities to generate income through Twitch strongly depend on these user statuses on the platform of which there are three stages that are passed through gradually: *standard*, *affiliate*, and *partner*. The standard status marks the default status and enables no (on-platform) monetization options. In contrast, the affiliate status opens up a few monetization opportunities, whereas only the partner status can fully operationalize all revenue sources. The decision on who is qualified to become affiliate or partner lies completely with Twitch. While the step to become an affiliate depends solely on measurable criteria (such as the number of followers or broadcasted minutes) and can be reached fairly quickly, receiving the partner status also includes subjective elements, which Twitch does not disclose any further (Twitch.tv, 2020b).^8^ Hence, achieving affiliate status is only the first step toward generating income from live streaming activities. Therefore, affiliates are more comparable to semi-professionals (who might augment their otherwise-derived income with revenues from streaming), while partners tend to spend their entire working time on streaming and derive the majority of their income from it.

This setting underlines the up-front investment streamers have to accept when they aim to professionalize. Therefore, to achieve a higher status and professionalize, one of the most important investments is that of time, not only as a certain amount of broadcast time per month is required (Twitch.tv, 2020a) but also because it takes time to build up an audience when starting a stream and even more so, an audience that tunes in regularly. The interviewees in Johnson and Woodcock (2019b) underline this reasoning, as they stressed the large amount of time that needs to be invested into building and maintaining a professional streaming career. The reasons for this are manifold: First, potential viewers have to notice that a streamer is online.^9^ Given the large competition streamers face, especially lesser known streamers without a large dedicated audience therefore profit from longer uninterrupted streaming sessions to accumulate higher viewer numbers, as typically, broadcasting streamers are displayed from large to small audiences on the website. Thus, an increase in time spent streaming is beneficial to fulfill the requirements for a status upgrade as well as reaching a larger audience, which subsequently increases the income potential.

Furthermore, time spent streaming should also directly correlate strongly with generated income as most of the revenue options presented in Table 1 are (heavily) dependent on live broadcasts. First, revenue from advertising clips can only be generated during broadcasts, as otherwise those clips will not be shown and the longer a stream, the more clips can be shown. Second, (virtual) currency transfers from viewers happen mostly during broadcasts. During broadcasts, a custom practice for streamers to encourage such “donations” is to acknowledge such them by saying a short “thank you” or reading out a short message sent with the donation. Additionally, many viewers use such transfers to acknowledge highly entertaining moments during a broadcast. Lastly, paid content production commissioned by third parties (e.g. for promotional activities) can obviously also only be realized when a streamer is actually broadcasting. Thus, total streaming time increases the overall success and income potential of a streamer. However, only in combination with a longer stream duration, an increase in total streaming time can unfold its full potential as e.g. ten one-hour sessions are likely to generate lower viewer attraction than two five-hour sessions.

### Identification strategy

Table 2 provides a short overview over our empirical setting while we lay out our arguments more deeply in the following paragraph. To measure activity on the platform, we focus on three indicators: streaming length, percentage of streams that occur on the weekend (in normal times, amateur and semi-professional users should have higher weekend activity) and total minutes streamed. Given that the different monetization options presented above are connected with a streamer’s user status, we expect streamers in these status groups to also be heterogeneous in their reaction to the changes brought by the pandemic. More precisely, we expect the decrease in opportunity costs to lead to an increase in streaming activity for some of the groups we establish, depending on their degree of professionalization, which we determine by their respective user status going into the pandemic (end of January 2020). Newcomer (standard) users with as-of-yet unrealized ambitions to professionalize have likely been more heavily constrained by other commitments making additional spare time critical to increase streaming activity, as these users e.g. need other jobs to earn a living. Additionally, the prime time for Twitch lies in the evening hours (Sullygnome, 2020), which is also the typical time frame for other social interactions with friends or family. Thus, we expect amateur newcomers to react most strongly to reductions in opportunity costs. Conversely, we would expect little to no reaction from streamers, who are already professionalized to a high degree (partners) as they already work from home and their lifestyles are accustomed to their streaming hours. If at all, they might be sensitive to the increase in overall viewership. However, we would expect their reaction to be very limited in scope due to an already high baseline of streaming activity and thus, a low marginal utility from any additional activity. Lastly, we would expect semi-professionals (affiliates) and experienced standard users to land with their reactions somewhere in-between the two other groups.

**Table 2:** Definition of Our Setting – Control, Treatment Groups and Periods

**Group/Period**	**Composition**	**Explanation**
Control	Established streamers with partner status based on our selection period (August 2019 – January 2020)	Partners have an array of monetization possibilities on Twitch and are expected to derive major parts of their income through their activity on the platform; we assume that they spend most of their working hours on Twitch and already operate on high capacity leaving little room for increasing efforts, so their streaming activities were least affected by the COVID-19 pandemic.
Treatment 1	Established streamers with either affiliate or standard status as of January 2020	Standard users and affiliates have, compared to partners, limited possibilities to earn money through their activity on the platform. As such, we assume that they spend their leisure time on Twitch. As the COVID-19 pandemic affected the time people spent at home, their streaming activities would be comparably more affected than those of our control group.
Treatment 2	Newcomer streamers, that newly appeared in the in our data collecting process after January 2020	As we started our data collecting in August 2019, we assume that streamers who first appeared in our data set in 2020 were newcomers to the platform that previously had not been able to generate (substantial) income and had not yet optimized their streaming behavior to a professional level, leaving comparatively more room to adjustments than the other groups. Especially in comparison to our first treatment group, they face larger uncertainty about their income potential on the platform and thus might be more inclined to increase their efforts to reveal said potential.
Pre-pandemic period	Week 5 (starting January 27) until week 10 (ending March 8) 2020	As we first observed streamer status during week 4, 2020, week 5 was the first complete week where we had data to categorize streamers into the aforementioned groups. Further, in the first weeks of 2020, available leisure time should have been affected by the end of the holiday seasons. As the strongest global pandemic reactions came into effect on or around March 14, 2020, which was at the end of week 11, we excluded that week from our regression sample.
Post-pandemic period	Week 12 (starting March 16) until week 29 (ending July 19) 2020	Week 12, 2020, marked the first complete week where societies and politics fully reacted to the pandemic (see Fig. 1). During July, for the first time in 2020 mobility levels reached those above pre-lockdown levels in all countries in Fig. 1. Since the pandemic situation started to show stronger differences between countries in summer, especially regarding work opportunities, our sample ended July 19, 2020.

In light of these considerations, our setting qualifies for a difference-in-differences (DiD) design, where we consider streamers holding the partner status prior to the pandemic as our control group, while the first treatment group consists of established streamers that had not (yet) reached partner status at that point in time (standard users and affiliates). Lastly, we define new users who entered the platform during the observed period as “newcomers” who make up our second treatment group.

Looking at potential effects through under- or unemployment, the established professional streamers (control group) should have remained unaffected in this regard, whereas less professionalized streamers should be comparably more affected due to larger increases in available time and potential need for (future) income. Especially in those cases, where streamers received unemployment benefits or short-time allowances, the opportunity costs for investing additional efforts into building a live streaming career should have been relatively low. In addition, for those who were not eligible for said benefits, the attractiveness of a professional career in a booming market such as live streaming has likely increased as well, as uncertainties about the duration of the pandemic and the recovery of “brick-and-mortar” businesses increased. Further due to their experience on the platform, the more established users (control and treatment group 1) likely have already found their utility-maximizing streaming efforts, while newcomers (treatment group 2) would not yet be settled and also face greater uncertainty regarding their income potential which they might overestimate due to the dominant presence of the most successful streamers. Thus, newcomers might try to take full advantage of their new situation with much more vigor than streamers who are already more accustomed to the platform. Given that live streaming is a very time-intensive task (Johnson & Woodcock, 2019b), newcomers might assume that the lack of time they were able to put into streaming was a crucial factor in limiting their prospects of becoming a professional streamer and thus should react comparatively stronger to a reduction in opportunity costs than our other groups.

## Data and descriptive statistics

### Data collection and curation

We base our analysis on a panel of streamers that were active in the first seven months of 2020. We first collected hourly, stream-level data from Twitch via its official API, covering the time range of August 2, 2019, and July 30, 2020. The resulting 3,329,042 observations include the top 500 live streams by current viewers at each hour of collection. We used this dataset as a starting point to obtain additional streamer-level data for each streamer who reached the hourly top 500 live streams at least once over the observed period, which required a minimum amount of 21 concurrent viewers. Thus, using only the top 500 live streams at first glance might give the impression of only covering the most successful streamers and that the resulting sample could thus suffer from survivorship bias, the low minimum viewer threshold of 21 shows that many streamers in our sample are far from what one would call successful. Furthermore, one single hourly top 500 placement of one single live stream already sufficed to be included in the sample, resulting in a final sample of 18,467 individual streamers. Additionally, the goal of this study is to gain insights on professionalizing behavior of streamers, which in the first place requires streamers to possess the intention for professionalization and at least some potential to be able to do so. Thus, while the Twitch platform boasts millions of regular streamers, since the entry barriers to initiating a stream are so low,^10^ active streaming alone does not signify any ambition to do so professionally. As such, including every single casual or even one-time streamer would likely result in a heavily biased sample.^11^

Subsequently, further data was extracted from data aggregator Sullygnome (Sullygnome, 2020), covering every unique stream initiated by each streamer in the panel, when available. This approach enabled us to retroactively collect the full activity history of streamers, even when their viewership numbers were too small to regularly enter the top 500 streams and allowed us to gather more accurate data on stream length and viewership.^12^ Additionally, we twice collected each observed streamer’s partnership status with Twitch, once on January 23rd and again on June 25th, 2020, to account for changes in partnership status.

As our interest lies in individual streamers, and many channels are run by a group of individuals or organizations who operate under different constraints, we performed further data cleansing. Using the given self-descriptions of each channel, we removed every channel that contained the words “studio” or “official,” as these words reliably indicate if a channel is operated by an organization. As Twitch does not extend partner status to channels specializing in gambling, even when all other requirements are fulfilled, we also excluded channels with descriptions containing the keywords “slots,” “casino” and “gambling.” Furthermore, we removed each stream with a duration longer than 24 h. Lastly, we ensured that all streamers in our sample streamed at least once in the immediate pre- and post-COVID-19 outbreak period, which we consider the six weeks leading up to and following the aforementioned cutoff. As calendar week 5 of 2020 marks the first complete week where data on the streamer status is present, we removed the first four weeks of 2020 as streamers could have held a lower status in these weeks. Besides, including these weeks would also risk biases in streaming activity due to the holiday season of 2019/2020. Thus, the first observations in the final sample started on January 27, 2020. Relying on the previously described changes in mobility and unemployment, we consider mid-March (calendar week 11) as the beginning of the post-COVID-19 outbreak period. This results in six pre-treatment weeks and 19 post-treatment weeks as the period of study. We chose a longer post-treatment period as we could simply continue our data collection process while the missing user status prior to January 27 prevents us from reliably using earlier data on streaming activity.

### Descriptive analysis

In total, the panel covers 1,936,528 unique streams initiated by one of the streamers within the panel. Table 3 provides the summary statistics of streams and stream characteristics in total and broken down by treatment and control groups for the whole period before and after the determined treatment period. As presented in Panel A of the table, all considered variables increased in the post-COVID-19 period except for the variable *percentage weekend*. Among the different stream components, the highest average growth was recorded by the variable total views (growth of 48.52%), while the growth of the other variables was below 20%. In Panel B, we differentiate the considered variables by control and treatment groups. On average, all variables (except for the weekend variable) for treatment group 2 are lower than for treatment group 1, which again are than for the control group. Thus, the differences in levels are just as we expected. Overall, growth rates in all variables are the highest for treatment group 2, indicating the strongest reactions. The highest absolute growth in total views and time streamed however can be observed in the control group.

**Table 3:** Descriptive Statistics of Streamer-level Measures of Total Sample and by Control and Treatment Groups

**Panel A: Total sample**									
		Pre-COVID-19				Post-COVID-19			
		Mean	Median	Min	Max	Mean	Median	Min	Max
N streams		25.46	24	1	314	28.93	28	1	284
Stream length		259.7	240.4	14	1,436.70	272.3	255.2	1	1,388.1
Total views		9,374	1,864	0	1,530,190	13,907	2,603	0	2,815,572
Time streamed		7,110	5,694	14	91.854	8,343	7,030	14	91,960
Percentage Weekend		0.279	0.27	0	1	0.278	0.27	0	1
N		18,467				18,467			

**Panel B: Sample means by control and treatment groups**									
	Control			Treatment 1			Treatment 2		
	pre	post	%diff	pre	post	%diff	pre	post	%diff
N streams	27.07	31.19	15.22	25.38	27.32	7.64	22.05	26.22	18.91
Stream length	286.1	298.2	4.22	256.7	264.3	2.97	205.9	226.5	10.0
Total views	17226	25010	45.19	2392	3624	51.5	1845	3841	108.18
Time streamed	8259	9716	17.64	6875	7581	10.27	4906	6384	30.13
Percentage Weekend	0.2688	0.2719	1.15	0.2831	0.2805	-0.9	0.2956	0.2862	-3.1
N	8,839			5,615			4,013		

Stream length and time streamed in minutes

**Fig. 3:**
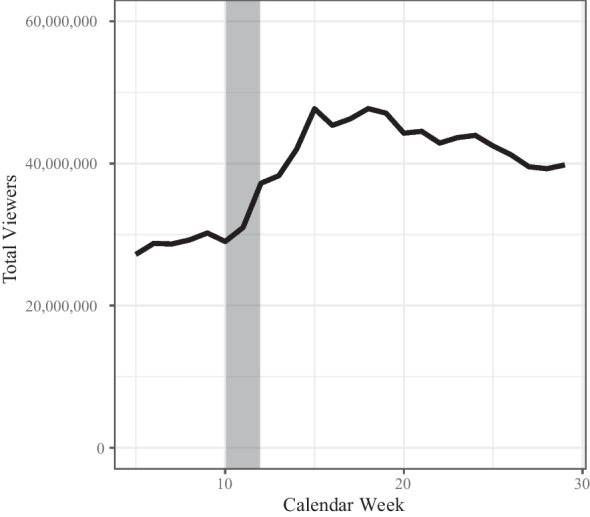
Plot of Total Weekly Viewership

**Fig. 4:**
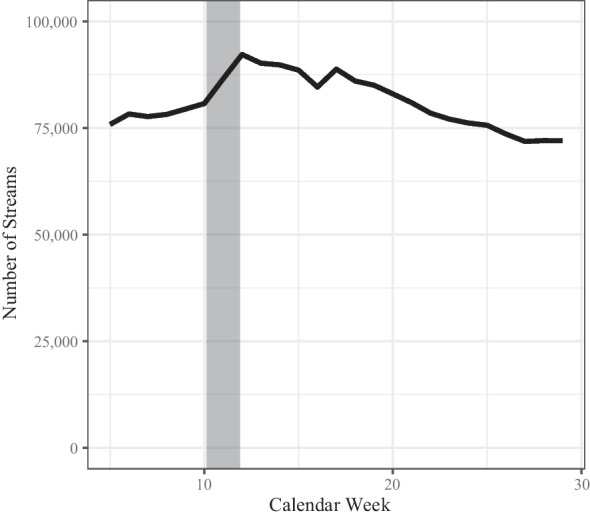
Plot of Total Weekly Streams

In Figs. 3 and 4, we show the viewership and stream activity (each aggregated by calendar week) over our observed period. Both time series show a stark increase after week 11, when the pandemic became a global phenomenon. Whereas the number of streams slowly returned to its previous level when, in the summer, COVID-19 case numbers were going down and several restrictions were lifted again, viewership remained on a higher level. This implies that overall, the streamers in our sample strongly benefitted from pandemic.

**Fig. 5:**
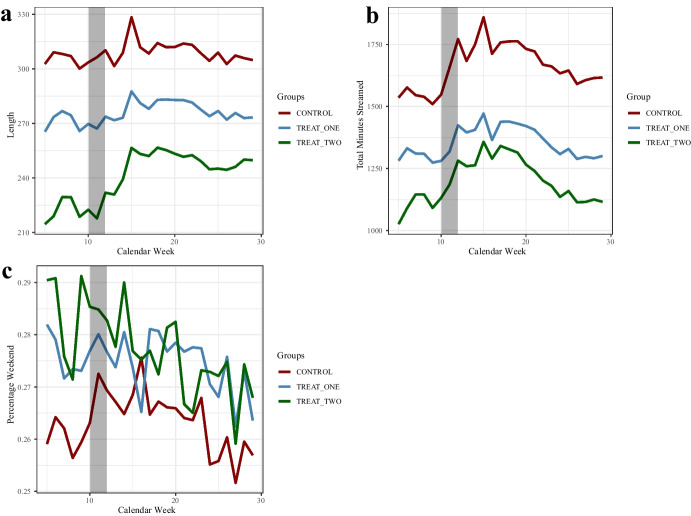
Stream Length, Total Minutes Streamed and Share of Weekend Streams over Time. Note: Grey bars mark week 11, when lockdown measures set in

In terms of group differences in streaming activity, plot a in Fig. 5 displays the average stream length per study group. Clearly, while all groups showed an initial positive reaction to the lockdown measures, the reaction of treatment group 2 was the strongest and stabilized at a much higher level than the other established groups. This further supports our assumption that the more established groups (control group and treatment group 1) had already found their utility-maximizing stream length, while newcomers were able to use the increased available time to increase their efforts and, hence, likely benefited more greatly from the sustainable increase in viewer numbers. Plot b then shows the total minutes streamed for each group. In contrast to plot a as well as our initial assumptions, next to the newcomers, professional streamers also showed a relatively strong reaction whereas that of treatment group 1 is much less pronounced. Although patterns for all groups look similar at first glance, newcomers and professional streamers both stabilized their total streaming time on a higher level than where they started. As streaming length did not change sustainably, it seems that professional streamers added further streams, whereas for newcomers instead increased their streaming length, perhaps even by doing fewer but longer streaming sessions. Lastly, plot c shows how the share of weekend streams changed over time. As assumed, the share of weekend streams is higher the lesser the professionalization of group and the downward path among the treatment groups implies that they increased streaming activity during the working week. However, the differences between the groups are relatively small and overall, the decrease was gradual rather than sudden.

## Empirical analysis

Using a DiD regression, we now estimate whether these trends significantly diverged after the pandemic unfolded. Our DiD approach will provide empirical evidence if the parallel trend assumption holds. This assumption assumes that without treatment, treatment and control groups follow similar trends in the dependent variable; i.e., that without the exogenous shock, the outcome variables of our three groups would have followed the same trend. We verify this assumption by illustrating the trend in our samples before the exogenous shock in Fig. 5: Similar trends before the treatment are indicated. When comparing the trends between the groups, one must keep in mind that the underlying dataset covers a global sample on a relatively frequent basis (weeks) in a highly dynamic market. If an overall common trend is visible despite differences in weather, time zones and potentially other country-specific factors, we argue that this suffices to fulfill the parallel trends assumption. For mean weekly streaming length and total minutes streamed, the parallel trends before treatment are clearly visible. For the share of weekend streams, the graphical evidence is less clear. Nonetheless, all groups show a significant downwards and upwards movement in a similar pattern before treatment sets in.

### Regression equation

We estimate the differences between streamers that responded more strongly to the exogenous shock (treatment groups) and streamers that were largely unaffected (control group), as explained in detail in "Methodology" section. Our formal DiD specification is written as follows:

1 $$\begin{aligned}{Y}_{it }=&\; \alpha + {\beta }_{1}*Pos{t}_{t} + {\beta }_{2} * Treat\,{One}_{i}+ {\beta }_{3} * Treat\,{Two}_{i }\\&+ {\beta }_{4 }*(Pos{t}_{t }* Treat\,{One}_{i }) + {\beta }_{5 }*(Pos{t}_{t }* Treat\,{Two}_{i }) \\&+ {\Gamma }_{i }+ {\Theta }_{t} + {\varepsilon}_{it}\end{aligned}$$

where the dependent variable ${Y}_{it}$ reflects streaming activity in terms of streaming length and total number of streams and stream percentage during weekends by individual $i$ in week $t$. $Pos{t}_{t}$ denotes a binary variable that equals one for post-lockdown periods and zero otherwise. The treatment dummies $Treat{ One}_{i}$ and $Treat{ Two}_{i}$ indicate whether a given individual belongs to one of the treatment groups and captures the differences between the three groups that exist irrespective of the lockdown. The interaction terms between the treatment and post-lockdown variables measure the difference between the groups in the post-lockdown period. If we assume that entrepreneurial ambition was higher for the treatment groups but followed a parallel trend prior to lockdown, then β_4_ and β_5_ capture the causal impact of these individuals experiencing a change in opportunity costs compared to established professional streamers who were largely unaffected by the pandemic on these points. Hence, for these coefficients we expect positive values. ${\Gamma }_{i}$ controls for geographic fixed effects (with spoken language acting as a proxy), and ${\Theta }_{t}$ captures time fixed effects.

### Main results

Table 4 provides the results of our DiD estimations. The coefficients of interest are the DiD estimators in rows four and five and confirm our graphical evidence. Column (1) shows the change in streaming length for treatment group 1 and treatment group 2 relative to the change in streaming length for the control group around the 25-week window surrounding week 11. The coefficients are positive and statistically significant; indicating that the post-COVID-19 period had an immediate effect on streaming length relative to partners. In particular, for treatment group 1 the estimate of 0.017 suggests that length was on average 1.7% higher in the post-pandemic period relative to the streaming length in the control group, whereas for treatment group 2, the post-period growth in streaming length was almost eight times higher with an estimate of 0.130 equaling to an additional increase of 13.0%.

**Table 4:** Effect of COVID-19 Pandemic on Streaming Behavior

Dep. Variables	(1)	(2)	(3)
	Log(length)	Pct Weekend	Log(Total Minutes)
TREAT_ONE	-0.179**	0.015**	-0.182**
	(0.002)	(0.002)	(0.007)
TREAT_TWO	-0.470**	0.024**	-0.481**
	(0.003)	(0.002)	(0.008)
POST	0.049**	-0.007**	0.088**
	(0.004)	(0.003)	(0.012)
POST × TREAT_ONE	0.017**	-0.005*	-0.066**
	(0.003)	(0.002)	(0.008)
POST × TREAT_TWO	0.130**	-0.012**	-0.001
	(0.004)	(0.003)	(0.010)
Constant	5.161**	0.280**	6.468**
	(0.007)	(0.004)	(0.0017)
Time FE	Yes	Yes	Yes
Language FE	Yes	Yes	Yes
Observations	1,936,528	375,391	375,391
R-squared (adj.)	0.047	0.003	0.049
F-Statistic	1,664.019**	21.907**	340.920**

Control group is defined as the subset of streamers who had the status of partner in January 2020. TREAT_ONE is defined as streamers who had the status of affiliate or standard in January 2020. TREAT_TWO is defined as streamers who entered the sample after January 2020 and thus had no pre-pandemic status. POST is defined as every week since week 12, 2020. Time fixed effects = Week FE. Robust standard errors in brackets

*p < 0.05; **p < 0.01

^a^*p* < 0.1

Although much smaller in effect size, we also find a 1.2 percentage points reduction in the share of weekend streams of treatment group 2 compared to the control group. Again, the reaction is less pronounced for treatment group 1 with 0.5 percentage points. Lastly, the results for total streaming time diverge from the previous pattern as well as our assumptions. In fact, treatment group 1 decreased their time spent streaming compared to the control group by 6.6%. For treatment group 2 on the other hand, the coefficient is not only close to zero, but also statistically insignificant, implying that this group behaved similarly to the control group.

**Fig. 6:**
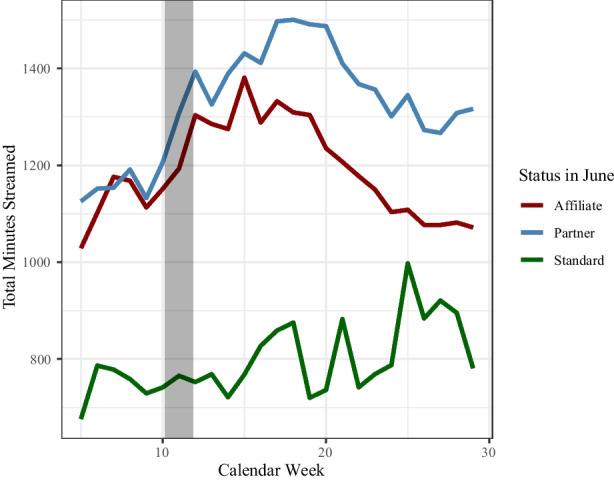
Total Minutes Streamed among Newcomers. Note: Newcomers were grouped by their acquired streamer status in June 2020. The gray bar marks week 11, when lockdown measures set in

Overall, these results show that newcomers in particular adapted their streaming behavior in response to the pandemic by making it more time-efficient (increasing in length per broadcast) and relocating into the working week (reduction in share of weekend streams). However, and especially in light of the other findings, the lack in difference to the control regarding total time spent streaming is puzzling. Still, there are two things that have to be kept in mind: First, the control group increased their streaming time stronger than we expected (possibly we underestimated the demand effect). Second, the more established streamers in treatment group 1 showed a negative and significant reaction, which implies that newcomers still reacted more strongly than treatment group 1 and on par with fully professionalized streamers. Thus, it seems there might be more to the story and hence we further investigate the newcomers’ changes total streaming time to get a better understanding of these results. It seems plausible that especially within this subgroup, there might be large differences depending on the market response for their increased efforts. Possibly, those newcomers who even managed to become partners in a short time reacted differently than those, whose efforts only lead to becoming an affiliate as such status changes strongly reduced their uncertainty regarding their own income potential from streaming. Figure 6 shows that those newcomers who reached at affiliate status by June 2020 initially increased their total streaming time to a similar degree as those who reached partner status. However, when in late spring case numbers were falling while lockdown policies were lifted and mobility increased again, new affiliates quickly started to reduce their streaming time down to pre-COVID-19 levels, while streamers who gained partner status stabilized their streaming time at a higher level. In contrast, users who remained standard users seemed to be completely unaffected (and are not considered in the following).

To assess whether the difference between affiliate and partner newcomers was significant, we estimated another DiD specification wherein the most successful group (new partners) was considered as the control group, while new affiliates made up the treatment group. The results are provided in Table 5. The POST dummy indicates that with the start of the pandemic, both groups increased their total streaming time by 22.7% which is almost three times larger than the 8.8% increase in our first estimation. Additionally and as expected from the visual analysis, newcomer affiliates streamed 15.4% less than newcomers who made the jump to the partner status.

**Table 5:** Effect of COVID-19 Pandemic on Streaming Behavior among Newcomers Who Reached Affiliate or Partner Status by June 2020

Dep. Variables	Log(Total Minutes Streamed)
TREAT	-0.098**
	(0.020)
POST	0.227**
	(0.037)
POST × TREAT	-0.154**
	(0.023)
Constant	6.303**
	(0.051)
Time FE	Yes
Language FE	Yes
Observations	66,786
R-squared (adj)	0.025
F-Statistic	34.087**

Subsample of streamers who entered the sample after January 2020 (newcomers). Control group is defined as streamers who had reached the status of partner by June 2020. TREAT is defined as streamers who had reached the status of affiliate by June 2020. POST is defined as every week since week 12, 2020. Time fixed effects = Week FE. Robust standard errors in brackets

*p < 0.05; **p < 0.01

^a^*p* < 0.1

## Discussion

The first wave of the COVID-19 pandemic led to a significant boost to activity on Twitch by both viewers on the demand side and streamers on the supply side. Regarding its differential effect on streamers by initial level of professionalization, we first confirmed our prior assumption: On average, a higher degree of professionalization is associated with a higher frequency and duration of broadcasts, as well as a larger share of streams conducted on weekdays. We then investigated how these measures changed in reaction to a decrease in opportunity costs while controlling for the degree of professionalization prior to the pandemic. We find that, by increasing the length of their broadcasts and shifting stream activity from the weekends towards weekdays, less professionalized streamers adapted their behavior to converge towards that of more strongly professionalized streamers, though this effect was only lasting for the least-professionalized group of newcomers. Interestingly, the most and least professionalized streamers initially increased their total streaming time more strongly than semi-professional streamers did. This is in contrast to our initial expectations, which were that streamers would be the less affected – and thus the less strong to react – the higher their initial degree of professionalization. An explanation for this might lie in the high degree of heterogeneity amongst even the most professionalized streamers (partners). Some of these partners might have been more affected by the social effects of the lockdown measures or the increase in viewership, which encouraged them to increase their streaming time, while others might not have been as professionalized as we had initially assumed.

Going back to our theoretical model of streaming as entrepreneurship, wherein an individual’s decision to increase professionalization efforts is responsive to decreases in opportunity costs (time, aversion to work effort, risk, income potential), it makes sense that whether increased efforts are sustained at a higher level or revert to pre-pandemic levels is dependent on market feedback. As a quick increase in status on the platform (or lack thereof) functions as a strong signal for income potential, such market feedback reduces individual uncertainty about the income potential and enables individuals to reevaluate the utility of their increased efforts. Given the relatively high time investment needed to build and sustain a live streaming career (Johnson & Woodcock, 2019b), marginal costs for any additional hour invested into professional streaming should be relatively high. Thus any increased effort needs to be compensated adequately (through direct earnings or potential future earnings) in order to be worth the investment. Newcomer affiliates and newcomer partners started on the same level of total streaming time and initially reacted in the same way. On the other hand, those newcomers who remained standard users neither showed a similar reaction, nor did they put in a large time investment in the first place.^13^ After four to eight weeks, however, it seems that the newcomers were able to better assess whether their increased efforts were indeed paying off and adapted their subsequent efforts accordingly with newcomer affiliates scaling back and newcomer partners remained on a higher level of time spent streaming. Initially, we assumed the affiliate status to be a steppingstone towards partner status, the results from the newcomer sample however imply that the majority of those streamers that achieved an increase in status must have become partners either directly or within a short timeframe of becoming affiliate.

Coming back to the broader scope of the Douglas and Shepherd’s (2000) model for career choice and the question why individuals with entrepreneurial ambitions still might prefer employment work we interpret our results as follows: An event like the pandemic changed individuals’ external conditions drastically in that some decided to actually “try their luck” to become entrepreneurs as they had relatively little to lose. In normal times, opportunity costs were simply too large to convert entrepreneurial intent to activity and individuals possibly felt more secure by engaging in other commitments (job, education, social). However, as successful newcomers sustained their increased activity on a higher level, our results point at the formerly unused potential that has been lying there and needed to be “awakened” by the pandemic.

In general, when – as a first step – individuals are able to try being an entrepreneur with relatively low risks, they are enabled to reduce their previous uncertainty and can subsequently reevaluate whether they want to continue pursuing entrepreneurship or prefer employment work instead. Such an environment caters especially to risk averse individuals and this is where digital entrepreneurship, but especially industries such as the content creation industry with its low entry barriers and large upside potentials can play an important role. As entrepreneurial activities in such industries can be conducted entirely from home and large parts of the involved costs are outsourced to the platforms, opportunity costs of time and income potential remain as the most relevant aspects to enable or prevent increased efforts. To keep a constant influx of new content creators and harvest the potential of groups such as our identified newcomers, platforms should therefore make sure to enable smooth paths into entrepreneurship. By supporting smaller content creators on their way into professionalization, platforms ensure that promising newcomers will not exit the platform prematurely (Bedingfield, 2022; Bernal, 2020; Klepek, 2022; Mellor, 2022; Twitch, 2019). Potentially, platforms could ease the way into professionalization and reduce the necessary time investment by devoting special areas for promising newcomers on their front page or put special emphasis on them in their recommendation algorithms as these typically cater to the most popular users. Note that this does not only apply to live streaming, as other areas of the content creation industry such as podcasts, or (development of) video games have similar characteristics and also saw increased demand and supply during the pandemic and especially from smaller content creators (Batchelor, 2020; Lee, 2020; VG Insights, 2021; VG Insights, 2022). Similarly, crowdfunding activity also increased dramatically during the pandemic (Chandler et al., 2021), which is also in line with Agrawal et al. (2018) who have shown that entrepreneurial activity rises during university breaks and now our study indicates similar results in another business area.

Based on our results and the idea of Douglas and Shepherd’s (2000) model for career choice, we further derive some ideas for policy implications to ease the way into entrepreneurship. Similarly to our ideas for digital platforms, local governments could try to provide easily accessible platforms to aspiring entrepreneurs. Such platforms could be both digital or physical, depending on the business ideas, e.g. pop-up store areas and digital marketplaces could be provided for small or even no fees for a limited amount of time. Another actual and successful example that relates to this idea is the “Club 100” project, which received large financial support from its regional government in Bremen, Germany. To support artists during the pandemic, the project provided a professional stage and infrastructure to perform live-streamed concerts and readings for an online audience and without the governmental support, the project would not have been economically profitable. Viewers had to buy tickets for the shows and the project was accessible for established artists as well as lesser-known newcomers (Dohme, 2021). The project was deemed as highly successful in supporting artists through the pandemic (HB People, 2021) and especially smaller artists benefited as the project was heavily featured in the local media and thus put these artists in a larger spotlight. Lastly, policymakers should acknowledge that platform work that may start as a hobby can also result in supplemental income generation, and ultimately in self-employment and entrepreneurship (which could generate further job opportunities for third parties). Thus, policymakers should keep these new opportunities in mind when designing tax laws or unemployment benefits to enable and not hinder smooth transitions through these stages.

### Limitations

This work is subject to several simplifications and, thus, limitations that we want to address. First, as we assigned individuals to the various treatment groups based on fairly unspecific, observable characteristics (their status on Twitch), there likely remains a high degree of heterogeneity within these broad groups. As previously argued, the partnered streamers, which we initially considered as close to fully professionalized, possibly consists to a significant degree of semi-professionals who we expected to be covered by the affiliate condition. The estimated differences in reaction between users on different professionalization levels should thus be considered closer to the lower bound of the true effect. Secondly, we do not account for specific lockdown policies in different countries in our analysis, nor do we consider different time zones. However, as we have shown in Fig. 1, changes in mobility were fairly comparable across the western world even though lockdown policies varied between and even within countries. Lastly, we are not able to differentiate whether the measured changes in streaming behavior are attributable to changes in either income or available time, as both factors were affected simultaneously. In order to differentiate between the two factors, more data on individual streamers’ employment background would have been necessary. Future research could pick up on that and take a deeper look at streamer-level data and also provide additional insights into the motivations of streamers as well as the determinants of success on the platform.

## Concluding remarks

In this study, we classify content creation and in particular live streaming as a form of entrepreneurship that is characterized by low financial costs and low entry barriers. In this context, we build upon the utility maximization model for career choice by Douglas and Shepherd (2000) to stress the relevance of opportunity costs as the most relevant factors for individuals to decide for or against increasing their own entrepreneurial efforts in this setting. We then evaluate whether and how individual streaming activity changes in response to a sudden disruption in opportunity costs such as time and income. For this, we consider the first wave of the COVID-19 pandemic that started in March 2020 as an exogenous shock mainly to available time and income and use status categories within the platform to differentiate between control and treatment groups. In summary, we find an increase in professional streaming activities in response to the start of the pandemic. Our DiD estimates indicate that a reduction in opportunity costs was followed by an increase of up to 13% in average stream length compared to our control group plus an additional 4.9% increase across all groups. Further, our results indicate a reduction of up to 1.2% in the share of weekend streams. Both variables indicate that professionalization efforts expanded to varying degrees, depending on the streamers’ status prior to the pandemic, when opportunity costs were lowered, especially for individuals with larger ex-ante uncertainty. Further, the ex-post analysis of newcomers’ success shows that when mobility increased beyond pre-pandemic levels and unemployment rates started to recover (due to re-openings in the hospitality and tourism sectors), the most successful newcomer streamers remained their efforts on a much higher level than prior to the pandemic. These newcomers have seemingly been able to transform their initial streaming efforts into sustainable (long-term) income solution, indicating that the reactions are indeed a result of the intention to pursue entrepreneurial efforts.

Our study thus sheds light on the large potential that lies in individuals who might be willing to become entrepreneurs but currently are satisfied in being employed. As they offer high flexibility and scalability, digital platforms therefore have the potential to cater to such individuals. In this context, the Douglas and Shepherd (2000) model provides a fitting framework according to which this potential could be “harvested” by changing outside conditions such as opportunity costs in favor of the entrepreneurship options. As most platform work and especially content creation has very low entry barriers, initial financial investment risks are relatively low, which should benefit risk-averse individuals in particular. Instead of being restricted by such downside risks, we identify opportunity costs, such as available time as the most constraining factors. Reducing opportunity costs should therefore be a key challenge for platform providers, as this ensures a steady influx of new content creators to keep the platforms attractive for other users.

Thus, our study contributes to the literature on entrepreneurial activity and opportunity costs, focusing especially on costs other than the common approach of using only foregone income (Agrawal et al., 2018; Burtch et al., 2018). Furthermore, the study also contributes to the literature on digital entrepreneurship (Kraus et al., 2019; Nambisan, 2017) and the emerging research on the entrepreneurial aspects of content creation (Ashman et al., 2018, Giertz et al., 2020; Johnson & Woodcock, 2019a, b; Mardon et al., 2018; Törhönen et al., 2020, 2021).

## Data Availability

Data and material is available from the corresponding author upon request.

## Appendix

**Table 6:** Overview of Live Streaming Platforms

Period	February 2020		June 2020	
Platform	Total hours watched (in hours)	Market share (in %)	Total hours watched (in hours)	Market share (in %)
Twitch	979,968,010	63,59	1,553,283,931	66,82
YouTube Gaming Live	351,522,493	22,81	471,578,917	20,29
Facebook Gaming	183,858,030	11,93	268,801,339	11,56
Mixer	25,611,913	1,66	30,997,472	1,33

Source: Data derived from Streamlabs (2020a, b)

**Table 7:** Composition of Initial Sample

	Count			Movement to			
Status	January	June	Net Change	Standard	Affiliate	Partner	Dropout
Standard	801	2,667	1,866	451	177	118	55
Affiliate	5,473	8,362	2,889	4	4,331	1,073	65
Partner	9,514	12,076	2,562	50	9	9,418	37
Newcomer	12,295	4,978	-7,317	2,162	3,845	1,467	4,821
∑	28,083	28,083	0	2,667	8,362	12,076	4,978

“Movement to” refers to changes in partnership status during January and June 2020

## Footnotes

1 Which in principle only consist of an internet connection and PC or smart device.

2 In their study, the authors use the terms self-employment and entrepreneurship interchangeably as they discuss the option of becoming an entrepreneur in contrast to performing employment work, even though e.g. Uber drivers can be considered as self-employed while it remains debatable whether their work can also considered to be entrepreneurial.

3 We will explain the practice of generating income through content creation later in "Methodology" section.

4 Twitch is by far the dominant leader in the market for live streaming. For a comparison with other market participants during our observation period see Table 6 in Appendix.

5 The perception of streaming as a viable source of income is also supported by recent surveys among young people who now regularly name *professional streamer* or *influencer* as one of their top dream jobs (Bruce, 2021; Skeldon, 2019).

6 E.g., Instagram had no paid subscription features for popular content creators for a long time, but has recently started to test such features (Instagram, 2022; Perez, 2022).

7 In the digital content creation industry, the term donation is typically used for voluntary monetary gifts from consumers. For these gifts, consumers typically cannot expect any service from the content creator in return. Still, the practice is often encouraged with the narrative of supporting the content creator and helping to ensure further content creation in the future. Thus it should not be confused with the typical definition of charity-related donations.

8 As of today, to be invited for affiliate status, streamers need to have at least 50 followers as well as to have broadcasted more than 500 min in total, on seven unique days, with an average of three or more concurrent viewers in the last 30 days (Twitch.tv, 2020a). Before applying for partner status, streamers have to fulfill further requirements (Twitch.tv, 2020b).

9 Which is why popular streamers typically start their broadcast with a countdown to that lets them appear as *live* on the platform and notify their followers with some lead-time to tune in. This helps to let the channel rise in Twitch’s recommendation algorithm or on the front page without requiring an immediate performance by the streamer. Smaller streamers typically lack a dedicated fan base that would make this strategy viable, thus longer stream durations are a crucial element to get noticed on Twitch and accumulate viewers.

10 E.g., Twitch streaming has been supported on every single PlayStation console starting in 2013 and can be enabled via a single button press.

11 The behavior of standard users in Fig. 6 most likely gives an impression on how this bias would affect our sample if probably millions of even smaller streamers were included in our sample.

12 In Table 7 in Appendix, we provide an overview of the initial sample composition.

13 Which leads us to believe that these users never had any real intention for professionalized streaming in the first place and possibly ended up in one of our top 500 observations by pure luck.

## Code availability

Code is available from the corresponding author upon request.
